# The Book World of Medicine and Science

**Published:** 1898-11-19

**Authors:** 


### The Book World of Medicine and Science.

Methods of Staining the Nervous System. By Dr. Bernhard Pollack. Translated by W. R. Jack, M D. (Glasgow: F. Bauermeister. 1898. Pp. 138. Price 4s.)

This is a translation from the German of Dr. Pollack's most successful treatise. It deals solely with the technique employed in microscopical examination of the nervous system, and does not refer to appearances presented under the microscope by prepared section. It is, in fact, a handbook for laboratory experts only; to them it cannot fail to be of great value. Almost every process of repute is mentioned, and formulæ and directions are given in great detail. Still, as the author in his preface states, "a certain amount of previous knowledge and experience has been taken for granted." The result is that the better known processes and methods are dealt with somewhat inadequately. It would have been better to have described them in full or not at all. The enormous strides that have been lately taken in neuropathology have rendered some such book as this a necessity; and to asylum pathologists in particular Dr. Pollack's book may be cordially recommended. The work of translation has been well and carefully done. Dr. Jack's style, too, is easy and unembarrassed. We have noticed but one slip, the confusion of photo-micrography with micro-photography, a very different matter.

Wright's Improved Physician's, Surgeon's, and Consultant's Visiting List for 1899. Compiled by Robert Simpson, L.R.C.P., L.R.C.S. Sixth edition. (Bristol: John Wright and Co.)

Among the various visiting lists which are annually offered to the profession this is certainly one of the best, if not actually the best, for daily use by the practitioner. The special feature by which it is chiefly distinguished from other such productions is that by a special arrangement of the leaves it is found possible, without increasing the width of the page, to make one opening and one entering of the names of the patients do for a month instead of for the usual week. Another peculiarity which, although a little thing, greatly adds to its utility, is the fact that the lines for the patients' names are printed alternately red and blue, which renders it far easier to run the eye along them than is the case when they follow one another in monotonous succession in the same colour. Practically, the closely-set lines have all the clearness of lines set at double the distance apart. In addition to the main part of the book, which, of course, is devoted to the "list," there are many useful tables and plenty of pages arranged for special purposes, such as cash, consultations, vaccinations, obstetric engagements, &c. This visiting list is issued in three forms—one the ordinary form for 1899; one which is called the perpetual list, which can be begun at any time of the year, an arrangement peculiarly suitable for growing practices; and another form specially prepared for the use of surgeons under the Factory and Workshops' Act.

A Text book of Zoology. By H. G. Wells, B.Sc., and A. M. Davies, B.Sc. (London: W. B. Clive. Pp. 358. Illustrated. Price 4s. 6d.)

This book, issued under the auspices of the University Correspondence College, is really the zoological portion, rewritten, of Mr. Wells' "Biology," published some five years ago. To attempt to review books such as this is not a little saddening. This particular book is not a text-book of zoology at all. It is a nicely-tabulated compendium of just so much as will "pass" any student of fair retentive powers through the zoological part of the Preliminary and Intermediate Science Examinations with least trouble. The book is made subservient to the Burlington House syllabus. Every London student knows what this means; the introduction to the rabbit, the hasty scamper through with amphioxus, astacus, and the rest. Of its sort the book is good; that it will serve the desired, but not desirable, end we have no doubt. But the "type system" is for personal teaching and not for compression into books of this class. When teachers such as we have had in London direct a class, students learn their science, and pass their examinations, too, without any need for resort to "tutorial books." And an interest is acquired which leads them to refer to the master-books of science, and to gain knowledge for the love of it. But the student who begins with tutorial works ends there. As we have said, this book is, in its way, good; the information is accurate, the diagrams are clear, and the language sufficiently simple and direct. But it is the publication of these "tutorial works" which suggests the strongest of all arguments for a reconstitution of the present University of London.

The Unconscious Mind By A. T. Schofield, M.D., M.R.C.S. (London: Hodder and Stoughton. 1898. Pp. 435. Price 7s. 6d.)

This book is written—so the author tell us—to establish the fact of an unconscious mind in man, and to trace in brief some of its powers and the various ways in which they are exhibited. But Dr. Schofield, though he has produced an interesting book, has not produced an important one. The author has undoubtedly forced not a few open doors; he has also vanquished more than one giant of his own imagining. The point of the whole psychological argument is indeed purely a verbal one. No one of any capacity will dispute the facts Dr. Schofield brings forward, examples of which are within the scope of our everyday experience. Every psychologist of standing has admitted inferentially or directly the workings of what Dr. Schofield calls the unconscious mind. The real point at issue between the authorities which Dr. Schofield so freely quotes—and it is a point which Dr. Schofield quite overlooks—is the purely verbal quibble as to whether these processes and workings should be referred to as mental or not. And it is a point settled, for each disputant, by his particular metaphysical bias. The materialist and the Haeckelian monist and the Jacksonian "parallelist" declare that these workings of the "unconscious mind" are the results simply of functional activity of the brain unaccompanied by consciousness. The idealistic and spiritualistic thinkers who refuse to take notice of the "physical series," recognising that these processes are unconscious, take refuge in a phrase —that of unconscious mentality." No one disputes the facts. And it is on the facts that Dr. Schofield bases the teachings which take up the second half of his book. It is certainly important to recognise, as Dr. Schofield does, the value of "unconscious education" in childhood. That is, to recognise the powerful, if unobtrusive influence of environment in moulding taste and character. But in this matter Dr. Schofield is sometimes a little inconsistent. He seems to find it difficult to decide whether education is justly a means of developing individuality or of repressing mental eccentricities. If the former, it may lead through eccentricity to madness, and hence much may be said for the repressive discipline of old-fashioned parents. If the latter, it may lead to the production of excellent but dull mediocrities. Some of the later chapters of the book are devoted to faith healing and similar perversities. The connection of these practices with the main thesis of the book is, perhaps, a little forced. But the conclusions of the author are eminently scientific. It is, however, a little hard to believe that Dr. Schofield cannot see that his conclusions, if correct, quite destroy the force of theological appeals to the evidential value of certain miracles. Such a result of his arguments, we are sure, Dr. Schofield did not anticipate when he wrote this book.

